# Pharmacokinetics and Pharmacodynamics of Tildipirosin Against *Pasteurella multocida* in a Murine Lung Infection Model

**DOI:** 10.3389/fmicb.2018.01038

**Published:** 2018-05-18

**Authors:** Dongping Zeng, Meizhen Sun, Zhoumeng Lin, Miao Li, Ronette Gehring, Zhenling Zeng

**Affiliations:** ^1^National Reference Laboratory of Veterinary Drug Residues, Laboratory of Veterinary Pharmacology, College of Veterinary Medicine, South China Agricultural University, Guangzhou, China; ^2^Institute of Computational Comparative Medicine, Department of Anatomy and Physiology, College of Veterinary Medicine, Kansas State University, Manhattan, KS, United States

**Keywords:** tildipirosin, pharmacokinetic/pharmacodynamic (PK/PD), murine lung infection model, *Pasteurella multocida*, minimum inhibitory concentration (MIC)

## Abstract

Tildipirosin, a 16-membered-ring macrolide antimicrobial, has recently been approved for the treatment of swine respiratory disease and bovine respiratory disease. This macrolide is extensively distributed to the site of respiratory infection followed by slow elimination. Clinical efficacy has been demonstrated in cattle and swine clinical field trials. However, the pharmacokinetic/pharmacodynamic (PK/PD) index that best correlates with the efficacy of tildipirosin remains undefined. The objective of this study was to develop a PK/PD model following subcutaneous injection of tildipirosin against *Pasteurella multocida* in a murine lung infection model. The PK studies of unbound (*f*) tildipirosin in plasma were determined following subcutaneous injection of single doses of 1, 2, 4, 6, and 8 mg/kg of body weight in neutropenic lung-infected mice. The PD studies were conducted over 24 h based on twenty intermittent dosing regimens, of which total daily dose ranged from 1 to 32 mg/kg and dosage intervals included 6, 8, 12, and 24 h. The minimum inhibitory concentration (MIC) of tildipirosin against *P. multocida* was determined in serum. The inhibitory effect I_max_ model was employed for PK/PD modeling. The area under the unbound concentration-time profile over 24 h to MIC (*f*AUC_0-24 h_/MIC) was the PK/PD index that best described the antibacterial activity in the murine infection model. The *f*AUC_0-24 h_/MIC targets required to achieve the bacteriostatic action, a 1-log_10_ kill and 2-log_10_ kill of bacterial counts were 19.93, 31.89, and 53.27 h, respectively. These results can facilitate efforts to define more rational designs of dosage regimens of tildipirosin using classical PK/PD concepts for the treatment of respiratory diseases in pigs and cattle.

## Introduction

Bovine respiratory disease (BRD) is the most common and costly disease of feedlot cattle in the world (Snowder et al., 2006; Gay and Barnouin, 2009). The infection usually involves stress from transportation, fatigue, anxiety, or viral infections combined with one or more bacterial pathogens (Rose et al., 2012). The bacterial agents most commonly linked to BRD are *Mannheimia haemolytica, Pasteurella multocida, Histophilus somni*, and *Mycoplasma bovis* (Snowder et al., 2006; Watts and Sweeney, 2010). Swine respiratory disease (SRD) constitutes some of the most important diseases of growing pigs and results in substantial economic loss and reduced welfare (Sørensen et al., 2006). Although these infections can be treated with one of several groups of antibiotics, effective treatment of these bacteria may be challenging because of antimicrobial resistance and limited therapeutic options (Lockwood et al., 2003; Godinho et al., 2005; Magstadt et al., 2018).

Tildipirosin is a semi-synthetic derivative of the naturally occurring 16-membered macrolide tylosin that has marketing authorizations for the treatment and prevention of BRD and treatment and metaphylaxis of SRD (EMA, 2011; Rose et al., 2016). The pharmacokinetic characteristics of tildipirosin have been studied in cattle and pigs (Menge et al., 2012; Rose et al., 2013; Torres et al., 2016). This macrolide is rapidly absorbed and extensively distributed to the site of respiratory infection followed by slow elimination. Clinical efficacy has been demonstrated for tildipirosin in cattle and swine clinical field trials (EMA, 2011; Bartram et al., 2016; Confer et al., 2016; Teixeira et al., 2017). The MIC_90_ values of tildipirosin against bacterial pathogens most commonly associated with BRD were determined as 1 μg/mL for *M. haemolytica* and *P. multocida* and 4 μg/mL for *H. somni* in different European countries (EMA, 2011). However, the pharmacokinetic/pharmacodynamic (PK/PD) index that best correlates with the efficacy of tildipirosin remains undefined. For macrolides such as tulathromycin, gamithromycin, and tildipirosin, good clinical efficacy and bacteriological cure are commonly achievable with plasma concentrations that are lower, even much lower, than *in vitro* MICs for major lung pathogens (Nowakowski et al., 2004; Huang et al., 2010; Menge et al., 2012; Toutain et al., 2017). Recently, it has been shown that free tulathromycin concentrations in serum suffice to explain the efficacy of single-dose tulathromycin in clinical use and a rational dosage regimen can be computed for macrolides (e.g., tulathromycin) using classical PK/PD concepts (Toutain et al., 2017).

In the present study, we determined the PK of unbound tildipirosin in a neutropenic murine lung infection model infected with *P. multocida*. We also evaluated the impact of dose and dosing regimens on the *in vivo* drug efficacy. The objectives of our studies were to elucidate the PK/PD index of tildipirosin that correlates best with efficacy against *P. multocida* in a murine lung infection model and to determine the target values of the PK/PD index to achieve various killing effects.

## Materials and Methods

### Antibiotic and Bacteria

Tildipirosin (40 mg/kg solution for subcutaneous injection) was obtained from HVSEN Biotechnology (Hubei, China). Tildipirosin (99.9% purity) was purchased from Sigma-Aldrich (St. Louis, MO, United States). *P. multocida* strain CVCC1669 (type B, serotype 2) was purchased from the China Veterinary Culture Collection Center (Beijing, China). The organism was grown, sub-cultured and quantified in Mueller-Hinton Broth (Becton Dickinson, Sparks, MD, United States) and Tryptic Soy Agar (Huankai, Guangzhou, China) supplemented with 5% defibrinated sheep blood (Puboxin Biotechnology, Beijing, China).

### Animals

Six-week-old, specific-pathogen-free (SPF), female ICR mice (weight: 22–24 g) were purchased from Guangdong Medical Laboratory Animal Center (Guangzhou, China). The mice were acclimatized for 1 week under SPF environmental conditions (20∼26°C, 40–70% relative humidity) before the experiment in the Laboratory Animal Center of South China Agricultural University (Guangzhou, China). The mice were supplied with SPF feed and water *ad libitum*. All procedures were approved by the Institutional Animal Care and Use Committee (IACUC) of South China Agricultural University (Approval No. 2016-A028) and were in accordance with the American Association for Accreditation of Laboratory Animal Care (AAALAC) guidelines.

### *In Vitro* Susceptibility Testing

The minimum inhibitory concentration (MIC) of tildipirosin against *P. multocida* CVCC1669 was determined in serum by broth microdilution methodology according to the Clinical and Laboratory Standards Institute (CLSI) recommended methods and quality control requirements. Susceptibility testing was performed in triplicates and mean value of MIC was used for data analysis.

### Murine Lung Infection Model

Neutropenia (neutrophils, ≤100 mm^3^) was induced via intraperitoneal injections of cyclophosphamide (Aladdin, Shanghai, China) for 4 days at 150 mg/kg of body weight and for one more day at 100 mg/kg of body weight prior to experimental infection. Lung infection was produced by using an intratracheal injection of 50 μL of an early-logarithmic-phase bacterial suspension [∼10^8^ colony forming units (CFUs)/mL] with a tracheal cannula under anesthesia with pentobarbital sodium as previously described (Dudhani et al., 2010; Qu et al., 2015). The bacterial suspension was introduced through the glottis via a 22G “Y” type intravenous catheter. When the catheter was successfully inserted into the trachea, the bacterial suspension was injected instantaneously. Thereafter, the animals were held in a vertical position for 15 s. Tildipirosin treatment commenced 2 h after inoculation, when an infection was reproducibly established (bacterial burden was 5.91 ± 0.56 log_10_CFU/lung).

### Pharmacokinetics of Tildipirosin in Neutropenic Infected Mice

Single-dose PK studies of tildipirosin were performed with neutropenic lung-infected mice following subcutaneous administration (dose volume: 0.2 mL) of tildipirosin (1, 2, 4, 6, and 8 mg/kg). The sedation/analgesic procedure was performed by placing animals in an induction chamber with oxygen flow rate at 0.5–1.0 L/min and isoflurane vapor flow rate at approximately 3–5% for induction and then at approximately 1–3% for maintenance. Blood was sampled by retro-orbital puncture and placed into heparinized tubes per dose level at 0.033, 0.083, 0.167, 0.25, 1, 2, 4, 8, 12, 24, 36, 48 h after drug administration (*n* = 4 animals per time point). Individual animals were sampled three or four times. The total volume collected from individual animals was less than 10% of the total blood volume. The samples were centrifuged at 3,000 *g* for 10 min at 4°C and the plasma was stored at -80°C until analysis. The concentration of tildipirosin in each plasma sample was determined by a validated liquid chromatography–tandem mass spectrometry (LC–MS/MS) method (Rose et al., 2013), with minor modifications. Plasma samples (0.1 mL) were deproteinized with 0.45 mL acetonitrile followed by centrifugation at 3,300 *g* for 10 min at 4°C. The supernatants (0.2 mL) were transferred into glass tubes and diluted with 0.6 mL ultrapure water. The calibration range was 0.01–5 μg/mL. The intraday and interday precision levels varied from 1.9 to 6.1% and from 2.6 to 4.8%, respectively. The limit of detection (LOD) and limit of quantification (LOQ) were 0.005 and 0.01 μg/mL, respectively.

The values of unbound tildipirosin plasma PK parameters in the single-dose PK studies were obtained by non-compartmental analysis in Phoenix WinNonlin^®^ 7.0 (Certara, L.P., Princeton, NJ, United States). The following parameters (referenced to the protein binding of 17% in plasma as previously described) (EMA, 2010), including the elimination half-life (t_1/2_), the area under concentration-time curve from 0 to 24 h (*f*AUC_0-24 h_), the peak drug concentration (*f*C_max_), and the time of maximum concentration (*f*T_max_) were calculated. The corresponding unbound plasma concentration-time profiles after multiple dosage regimens were predicted using Phoenix’s non-parametric superposition function based on the single-dose unbound plasma PK concentration-time profile.

### Pharmacodynamics of Tildipirosin in Neutropenic Mouse Lung Infection Model

Neutropenic mice were infected with *P. multocida* CVCC 1669 2 h prior to the initiation of treatment. Group of two mice were treated for 24 h with 20 dose regimens included five dose levels (1, 2, 4, 6, and 8 mg/kg) administered subcutaneously using four dosing intervals (every 6, 8, 12, and 24 h). Total dose of tildipirosin ranged of 1–32 mg/kg/24 h. The mice were sacrificed after 24 h of therapy, and the lung samples were aseptically removed and homogenized for CFU determination. Untreated control mice were sacrificed before tildipirosin treatment and at 24 h after treatment.

### Data Analysis

The PK/PD analysis was conducted using the inhibitory effect I_max_ model (Riviere and Toutain, 2011). This model is described by the following equation:

$$\begin{array}{c} E=E_{o}-\frac{I_{\max}\cdot X}{IC_{50}+X} \end{array}$$

Where *E* is the antibacterial effect, measured as change in log_10_CFU/lung after 24 h of treatment, compared with the initial log_10_CFU/lung in the untreated control mice; *E_0_* is the difference in number of bacteria (log_10_CFU/lung) in control samples between time 0 and 24 h. *I_max_* is the maximum antimicrobial growth inhibition determined as the change in log_10_CFU/lung after 24 h treatment with tildipirosin. *X* is the predictive variable (express as *f*AUC_0-24 h_/MIC, *f*C_max_/MIC, and *f*%T > MIC), *IC_50_* is the *X* value producing 50% of the maximum antibacterial effect. The relationship between efficacy and the three PK/PD indices was determined by non-linear least-squares regression in Phoenix WinNonlin^®^ PD model. The coefficient of determination (*R*^2^) was used to estimate the variance due to regression for each of the PK/PD indices.

## Results

### *In Vitro* Susceptibility Testing

The average MIC of tildipirosin against *P. multocida* CVCC 1669 in serum was 0.25 μg/mL.

### Pharmacokinetics of Tildipirosin in Neutropenic Infected Mice

**FIGURE 1:**
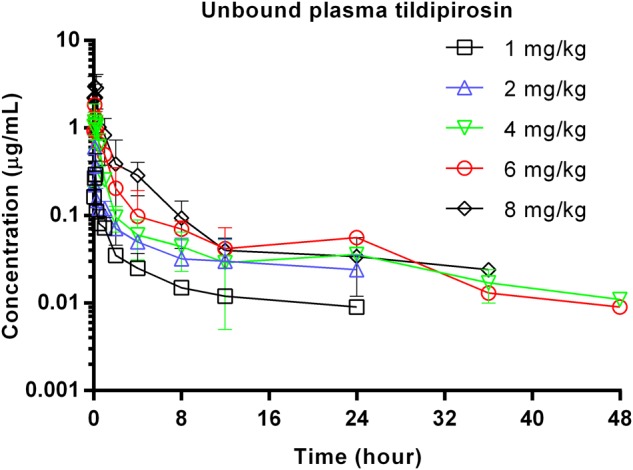
Unbound plasma tildipirosin concentration-time courses in neutropenic infected mice following single subcutaneous injections of 1, 2, 4, 6, and 8 mg/kg.

The unbound plasma tildipirosin concentration-time courses in neutropenic infected mice following single subcutaneous injection at 1, 2, 4, 6, and 8 mg/kg are shown in **Figure 1**. The plasma protein binding of 17% was employed to generate the corresponding time course profiles for unbound plasma tildipirosin, and the derived values of PK parameters are presented in **Table 1**. The *f*AUC_0-24 h_ and *f*C_max_ for the escalating single doses were increased in a dose-dependent manner, ranging from 0.49 to 4.14 μg⋅h/mL and 0.30 to 2.99 μg/mL, respectively. The elimination half-life ranged from 13.67 to 38.25 h.

**Table 1:** Pharmacokinetic parameters for unbound plasma tildipirosin concentrations following single subcutaneous injections (1–8 mg/kg) in neutropenic infected mice.

Parameter	Single subcutaneous injection level				
	1 mg/kg	2 mg/kg	4 mg/kg	6 mg/kg	8 mg/kg
*f*C_max_ (μg/mL)	0.30	0.60	1.16	1.84	2.99
*f*T_max_ (h)	0.17	0.08	0.08	0.08	0.08
T_1/2_ (h)	23.03	38.25	26.40	13.67	32.56
*f*AUC_0-24 h_ (μg⋅h/mL)	0.49	1.00	1.71	2.54	4.14

*f*C_*max*_: the peak unbound plasma tildipirosin concentration; *f*T_*max*_: the time of maximum unbound plasma tildipirosin concentration; T_*1/2*_: half-life; *f*AUC_*0*-*24h*_: the area under unbound plasma tildipirosin concentration-time curve over 24 h.

### Relationships Between PK/PD Indices and Antibacterial Activity

**FIGURE 2:**
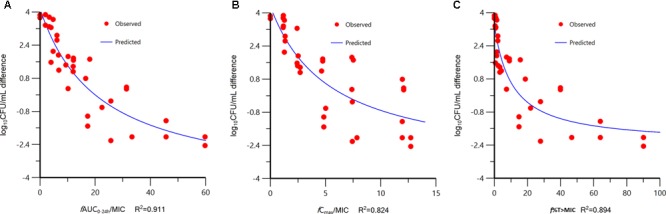
Relationships between the effect of tildipirosin against *Pasteurella multocida* CVCC 1669 and the PK/PD indices *f*AUC_0-24 h_/MIC **(A)**, *f*C_max_/MIC **(B)**, and *f*%T > MIC **(C)** in the murine lung infection model. Each symbol represents the datum from a single lung. *R*^2^ is the coefficient of determination.

At the start of tildipirosin therapy, the bacterial burdens were 5.91 ± 0.56 log_10_CFU/lung. In untreated animals, the organisms grew at a rate of 3.79 ± 0.07 log_10_CFU/lung over the next 24 h. The most effective tildipirosin dosage regimens result in reductions, relative to the bacterial number at the start of tildipirosin treatment, of 2.24 ± 0.21 log_10_CFU/lung. The relationships between the effect of tildipirosin against *P. multocida* and each of the PK/PD indices in the lung infection model are shown in **Figure 2**. The strongest relationships were observed when results were correlated with the *f*AUC_0-24 h_/MIC, with *R*^2^ value of 0.911 (*f*C_max_/MIC *R*^2^, 0.824; *f*%T > MIC *R*^2^, 0.894). The *f*AUC_0-24 h_/MIC ratios required for the various efficacy targets in the lung infection model are shown in **Table 2**.

**Table 2:** The PK/PD parameter estimates for the *f*AUC_0-24 h_/MIC index and the *f*AUC_0-24 h_/MIC values required for various antibacterial effects.

Parameter	Mean ±*SD*
*E_0_* (log_10_CFU/lung)	4.16 ± 0.43
*I_max_* (log_10_CFU/lung)	8.66 ± 1.10
*IC_50_*	21.57 ± 8.38
*f*AUC_0-24 h_/MIC for bacteriostatic action	19.93 h
*f*AUC_0-24 h_/MIC for 1 log_10_ kill	31.89 h
*f*AUC_0-24 h_/MIC for 2 log_10_ kill	53.27 h

## Discussion

Previous studies have shown that tildipirosin administered by single subcutaneous administration to cattle and pigs exhibits high bioavailability, rapid distribution to lung, and a long elimination half-life to provide therapeutic or preventive efficacy against BRD and SRD (EMA, 2011; Menge et al., 2012; Rose et al., 2013). These earlier studies suggest that further research is needed to gain deeper insights into the PK/PD relationship of tildipirosin. In the present study, we used a murine lung infection model to determine, for the first time *in vivo*, the PK/PD indices best predictive of activity against *P. multocida* and the magnitude of the predictive indices required for various magnitudes of killing effect. Consistent with other macrolides (Modric et al., 1998; Toutain et al., 2017), tildipirosin dose fractionation experiments demonstrated that the *f*AUC_0-24 h_/MIC ratio was most closely linked to the therapeutic efficacy against *P. multocida*.

Following subcutaneous administration, tildipirosin is rapidly absorbed in mice as demonstrated by the times reaching maximum plasma concentrations observed at as early as 5 min after administration in this study, which was significantly faster than the 23 min observed in bovine (Menge et al., 2012). Both absorption and overall exposure to tildipirosin, reflected by *f*C_max_ and *f*AUC_0-24 h_, respectively, in *P. multocida* infected mice displayed dose proportionality over the dose range from 1 to 8 mg/kg. The non-parametric superposition principle was applied to the single-dose unbound plasma tildipirosin concentration-time curves to generate the unbound plasma concentrations for the multiple dose regimens across the 24 h treatment period. The non-parametric superposition principle was based on the following assumptions: (1) each dose of a drug acts independently of every other dose; (2) the rate and extent of absorption and average systemic clearance are the same for each dosing interval; and (3) linear pharmacokinetics apply so that a change in dose during the multiple dosing regimen can be accommodated.

MIC_90_ values of tildipirosin against the pathogens most commonly involved in the etiology of BRD were determined as 1 μg/mL for *M. haemolytica* and *P. multocida* and 4 μg/mL for *H. somni* under standard CLSI testing conditions in epidemiologically unrelated bovine isolates (wild-type) collected in different European countries (EMA, 2011). Clinical efficacy has been demonstrated for tildipirosin in cattle and swine clinical field trials. However, similar to other macrolides such as tulathromycin, gamithromycin, and tilmicosin, the plasma concentration of tildipirosin after single intramuscular injection at 4 mg/kg body weight is clearly below the *in vitro* MIC_90_ levels for major lung pathogens (Shryock et al., 1996; Modric et al., 1998; Benchaoui et al., 2004; Nowakowski et al., 2004; Godinho, 2008; Huang et al., 2010). The described at least fourfold reduction in *A. pleuropneumoniae* MICs appears to be a conservative correction for *in vitro* activity when interpreting tildipirosin activity for *in vivo* pH and serum situations in swine (Rose et al., 2013). The *in vitro* susceptibility of Macrolides and ketolides show a considerable enhancement of antimicrobial activity against *P. aeruginosa* in RPMI 1640 medium and other eukaryotic media through increased uptake and reduced efflux (Buyck et al., 2012). More recently, the *P. aeruginosa* became susceptible when tested in a eukaryotic medium rather than a conventional broth, suggesting that measuring MICs in RPMI-1640 could be easily implemented to phenotypically detect acquired resistance to macrolides in *P. aeruginosa* from cystic fibrosis patients (Mustafa et al., 2017). Therefore, the crucial proviso is that MICs have to be determined in serum instead of artificial medium such as Mueller-Hinton broth, when establishing PK/PD relationships as a basis for macrolide dosage determination and prevention of resistance emergence (Lees et al., 2006; Toutain et al., 2017).

The PK/PD analysis revealed that *f*AUC_0-24 h_/MIC was the PK/PD index that was the most predictive of the antibacterial effect of tildipirosin against *P. multocida*. In our models, the *f*AUC_0-24 h_/MIC ratio appeared to be only slightly more predictive than *f*%T > MIC of *in vivo* bacterial killing, on the basis of the *R*^2^ values, although visual examination of the relationships revealed a relativity large scatter at *f*AUC_0-24 h_/MIC values of 20–40 (**Figure 2**). While most macrolides have been classified as time-dependent killing drugs, best described by the PK/PD parameter time above MIC (T > MIC), for newer macrolides such as azithromycin and clarithromycin, the plasma AUC/MIC ratio appears to be the best correlate with successful outcome (Lees et al., 2006). Similar observations in regard to the discrimination between PK/PD indices have been made by use of tissue cage model with tulathromycin (Toutain et al., 2017; Zhou et al., 2017). Those studies indicate that free tulathromycin concentrations in serum suffice to explain the efficacy of single-dose tulathromycin in clinical use and a dosage regimen can be computed for tulathromycin using traditional PK/PD concepts. DeDonder et al. (2016) investigated PK/PD of gamithromycin in pulmonary epithelial lining fluid (PELF) in naturally occurring BRD in multisource commingled feedlot cattle. The findings from that study indicate that PK/PD indices were predictive of positive treatment outcomes and a significant association was found between treatment success and PELF AUC_0-24 h_/MIC for *P. multocida*.

## Conclusion

This study is the first to demonstrate in murine lung infection model that *f*AUC_0-24 h_/MIC is the PK/PD index that is the most strongly linked to the antibacterial effect, and we determined the *f*AUC_0-24 h_/MIC targets in plasma for achieving various magnitudes of bacterial kill. Our study provides key pharmacological information for optimized clinical use of tildipirosin against infections caused by *P. multocida*.

## Author Contributions

ZZ conceived this study and participated in its design and coordination. DZ designed the experiments and drafted the manuscript. MS carried out the *in vivo* animal experiments. DZ, ZL, RG, and ML conducted the PK/PD analysis. All authors read and approved the final manuscript.

## Conflict of Interest Statement

The authors declare that the research was conducted in the absence of any commercial or financial relationships that could be construed as a potential conflict of interest.
